# Birth preparedness and complication readiness among women of reproductive age in Kenya and Tanzania: a community-based cross-sectional survey

**DOI:** 10.1186/s12884-020-03329-5

**Published:** 2020-10-19

**Authors:** James Orwa, Samwel Maina Gatimu, Michaela Mantel, Stanley Luchters, Michael A. Mugerwa, Sharon Brownie, Leonard Subi, Secilia Mrema, Lucy Nyaga, Grace Edwards, Loveluck Mwasha, Kahabi Isangula, Edna Selestine, Sofia Jadavji, Rachel Pell, Columba Mbekenga, Marleen Temmerman

**Affiliations:** 1 Centre of Excellence Women and Child Health/MERL, Aga Khan University, P. O. Box 30270–00010, Nairobi, Kenya; 2 Department of Population Health, Aga Khan University, Nairobi, Kenya; 3 School of Nursing and Midwifery, Aga Khan University, Nairobi, Kenya; 4 Department of Obstetrics and Gynaecology, Aga Khan University, Nairobi, Kenya; 5 Department of Public Health and Primary Care, Ghent University, Ghent, Belgium; 6 Department of Epidemiology and Preventive Medicine, Monash University, Melbourne, Australia; 7 Burnet Institute, Melbourne, Australia; 8 Aga Khan Health Services, Dar es Salaam, Tanzania; 9 Centre for Health & Social Practice, Waikato Institute of Technology (Wintec), Hamilton, New Zealand; 10 School of Medicine, Griffith University, Brisbane, QLD Australia; 11 Green Templeton College, Oxford University, Oxford, UK; 12 Ministry of Health, Community Development, Gender, Elderly and Children, Dar es Salaam, Tanzania; 13 Regional Reproductive and Child Health Office, Region, Mwanza, Tanzania; 14 School of Nursing and Midwifery, Aga Khan University, Kampala, Uganda; 15 School of Nursing and Midwifery, Aga Khan University, Dar es Salaam, Tanzania; 16 Aga Khan Foundation, Ottawa, Canada

**Keywords:** Birth preparedness, Complications readiness, Obstetric danger signs, Pregnancy, Kenya, Tanzania, Eastern Africa, BPCR, Safe motherhood, Maternal health

## Abstract

**Background:**

Delayed health-seeking continues to contribute to preventable maternal and neonatal deaths in low resource countries. Some of the strategies to avoid the delay include early preparation for the birth and detection of danger signs. We aimed to assess the level of practice and factors associated with birth preparedness and complication readiness (BPCR) in Kenya and Tanzania.

**Methods:**

We conducted community-based multi-stage cross-sectional surveys in Kilifi and Kisii counties in Kenya and Mwanza region in Tanzania and included women who delivered two years preceding the survey (2016–2017). A woman who mentioned at least three out of five BPCR components was considered well-prepared. Bivariate and multivariable proportional odds model were used to determine the factors associated with the BPCR. The STROBE guidelines for cross-sectional studies informed the design and reporting of this study.

**Results:**

Only 11.4% (59/519) and 7.6% (31/409) of women were well-prepared for birth and its complications in Kenya and Tanzania, respectively, while 39.7 and 30.6% were unprepared, respectively. Level of education (primary: adjusted odds ratio (aOR): 1.59, 95% CI: 1.14–2.20, secondary: aOR: 2.24, 95% CI: 1.39–3.59), delivery within health facility (aOR: 1.63, 95% CI: 1.15–2.29), good knowledge of danger signs during pregnancy (aOR: 1.28, 95% CI: 0.80–2.04), labour and childbirth (aOR: 1.57, 95% CI: 0.93–2.67), postpartum (aOR: 2.69, 95% CI: 1.24–5.79), and antenatal care were associated with BPCR (aOR: 1.42, 95% CI: 1.13–1.78).

**Conclusion:**

Overall, most pregnant women were not prepared for birth and its complications in Kilifi, Kisii and Mwanza region. Improving level of education, creating awareness on danger signs during preconception, pregnancy, childbirth, and postpartum period, and encouraging antenatal care and skilled birth care among women and their male partners/families are recommended strategies to promote BPCR practices and contribute to improved pregnancy outcomes in women and newborns.

## Background

Early preparation for childbirth throughout the continuum of care (pregnancy, delivery, and postnatal) is essential in preventing maternal and neonatal deaths [1]. Annually, 250,000 women die of pregnancy-related causes of the 30 million pregnant women in Africa [2]. Advanced preparation for birth is crucial in improving birth outcomes [3] and helps avoid delays in deciding where to seek maternal healthcare services, reaching a healthcare facility, and getting appropriate care upon reaching health facility [3]. Evidence shows that these three delays affect access to quality maternal care and contributes to poor maternal and neonatal health outcomes [4].

Reduction in neonatal mortalities is associated with birth preparedness and complication readiness (BPCR) interventions in developing countries [5]. In most countries, pregnant women receiving antenatal care (ANC) are counselled on birth plans and identification of danger signs during pregnancy, delivery, and postnatal period [5]. In Kenya and Tanzania, BPCR packages are provided as part of focused ANC services to reduce maternal and neonatal morbidity and mortality. However, the proportions of women who are well-prepared for birth in Eastern Africa is low. Only 22.3, 28 and 28.3% of women were well-prepared for birth in facility-based studies in Rwanda [6], West Pokot Kenya [7] and Uganda [8], respectively. While, 25.7–38.9 and 58.4% of pregnant women were well-prepared for birth in community-based studies in Ethiopia [1, 9–12] and Chamwino district in Tanzania [13], respectively. Besides, statistics show that 96 and 98% of pregnant women attend ANC at least once, and 62.5 and 62.2% attend at least four (4) ANC consultations in Kenya and Tanzania respectively [14, 15]. However, only 61.2 and 62.6% of the women deliver at a health facility in Kenya and Tanzania respectively [14]. ANC is an important factor in reducing maternal and neonatal mortality [16] and the World Health Organization recommends at least eight ANC visits during the pregnancy [17].

During birth preparedness counselling services at the ANC, women are encouraged to identify a place of birth for safe, skilled and emergency delivery care, birth attendant to increase the availability of skilled attendance and help reduce delays in obtaining care and blood donors in case of haemorrhage due to the persistent shortage of safe blood at most health facilities, arrange for transport to the care site and set aside money for the birth services and transport [6, 9, 13]. Only a few women are, however, prepared in at least three of these areas of birth plan preparations or could identify three or more danger signs across the continuum of care in Uganda [18], Ethiopia [19–21], Burkina Faso [22], and Tanzania [23]. In Tanzania, factors such as long distances from health facilities, difficulty in finding transport to health facilities, cost of transport and hospital bills, which have been linked to the high maternal deaths [24] are discussed during ANC visits as part of BPCR.

BPCR is associated with ANC [13], secondary and tertiary education levels [13, 25], young age of the mother [25], knowledge of danger signs [6, 10, 12, 13], delivery at a health facility [13, 26], and assistance from community health workers [6, 27]. ANC is associated with knowledge of obstetric danger signs [26] and regarded as a key facilitator of BPCR [28].

The low level of birth preparedness [7, 13], inadequate ANC attendance [14, 15], and persistent poor maternal high maternal mortality of 362 per 100,000 live births in Kenya [14] and 556 per 100,000 live births in Tanzania [15] and persistent challenges in accessing maternal healthcare [24] could highlight inadequate BPCR interventions in Kenya and Tanzania. Therefore, this study assessed the level of BPCR and its associated factors in Kenya and Tanzania among women who delivered in the 2 years preceding the study. The findings of the study will inform for the health planners and decision-makers both locally and nationally to identify appropriate interventions to improve BPCR of pregnant women and their families.

## Methods

### Study design and setting

The study used quantitative baseline data from the AQCESS (Access to Quality Care through Extending and Strengthening Health Systems) and IMPACT (Improving Access to Reproductive, Maternal and Newborn Health in Mwanza) projects, both executed by the Aga Khan Foundation Canada with funding from the Government of Canada, being implemented in Kenya and Tanzania, respectively. The projects aim to contribute to the reduction of maternal and neonatal mortality in Kisii (Bomachoge Borabu sub-county) and Kilifi (Kaloleni/Rabai sub-counties) counties in Kenya and Mwanza region (Illemela, Nyamagana, Buchosa, Sengerema, Ukerewe, Misungwi, Kwimba and Magu districts) in Tanzania. Bomachoge Borabu sub-county is one of the nine sub-counties in Kisii County with a population of 129,617 people. In 2015, the county had 53.3% of health facilities deliveries and 76.6% deliveries assisted by skilled providers [14]. Kaloleni and Rabai are coastal sub-counties in Kilifi County with a population of 304,778; 52.6% health facility deliveries and 52.3% skilled birth attendance [14]. Mwanza region lies in the northern part of Tanzania and has a population of 2,772,509 people; 57.2% of the women attending at least four ANC visits and 75.3% hospital delivery [29]. The two countries and the regions within the countries were chosen due to their high maternal mortality [14, 15].

### Sample size and sampling technique

The sample size for the households was calculated to detect a 10% difference in skilled birth attendance between the projects’ baseline and end line. The sample size was calculated using the proportion of deliveries assisted by a skilled provider for each of the study areas (61.8% for Kenya and 63.7% for Tanzania), design effect of 2, level of significance of 95%, a margin of error of 5% and non-response rate of 10%. The total number of households required for the survey were 960 in Kisii, 1100 in Kilifi, and 1676 in Mwanza. Out of which 518, 664, and 1176 women were eligible and interviewed in Kisii, Kilifi, and Mwanza, respectively. A community-based multi-stage cluster design was used. A total of 30 villages each in Kenya and Tanzania were selected based on the number of households in the first stage followed by a random selection of households from lists of households within the villages in the second stage. At the households, all women of reproductive age and who consented were interviewed.

### Data collection

Data were collected in August 2016 and August 2017 for AQCESS and IMPACT projects, respectively using a pretested questionnaire with questions about BPCR adopted from the monitoring BPCR tools for maternal and newborn health was used for data collection [30]. The English questionnaire was translated into Swahili, Ekegusii and Kigiriama; the common languages among the study participants. Trained data collectors entered data to the Open Data Kits platform which had electronic versions of the questionnaires with in-built data validation and quality checks. Data were stored onto a secure cloud server after a completeness check by a supervisor. All the selected households were included in the interviews; in case there was no eligible respondent available at the time of data collection, three revisits attempts were made before the households were declared unavailable.

### Study variables

BPCR, the main outcome variable, was assessed by asking women if a member of their family or herself prepared the following on the last birth: “1*) discuss the place of delivery, 2) discuss who will perform the delivery, 3) set aside funds for the delivery, 4) arrange transport, and 5) identify a blood donor*.” A woman was considered to be *well-prepared* for birth and its complications if she mentioned at least three out of five key components of BPCR [6, 9, 13], and *less prepared* if mentioned less than three and *not prepared,* if she mentioned none. Similarly, a woman was considered to have *good knowledge* about danger signs if she spontaneously mentioned at least three danger signs during pregnancy, labour and childbirth and postpartum [2, 31]; *poor knowledge,* if she mentioned less than three, and *no knowledge,* if she mentioned none. A list of all danger signs in each continuum of care is included in additional file 1.

Independent variables included maternal age, level of education (none, primary, secondary+), place of delivery (home, health facility), number of ANC visits (none, 1–3 visits, 4+ visits), and knowledge of danger signs during pregnancy, labour and childbirth, and postpartum.

### Statistical analysis

Categorical data were described using frequencies and percentages and continuous data using median and interquartile ranges (IQR). A univariate model was fitted to examine associations between each variable and the ordered categories of BPCR. Variables with *p*-value < 0.25 in the univariate model were fitted in the multivariable Proportional Odds regression model to determine their association with the dependent variable (ordered categories of BPCR) while controlling for the confounding effect of the explanatory variables [32].

Due to the ordinal nature of the outcome, Proportional Odds model [32] with a logit link function was used in both univariate and multivariable regression analysis to determine the association between the explanatory variables and the outcome. For the three categories of the outcome, the response is equivalent to two binary responses; (i) well-prepared versus less prepared or not prepared and (ii) well-prepared or less prepared versus not prepared. In this case, there is a cut-off point (threshold) at well-prepared the first logit and another at less prepared to form the second logit. The model can be defined in its simplest form as follows:

1 $$ logit\left[P\left(Y\le j|\boldsymbol{x}\right)\right]={\alpha}_j+{\boldsymbol{\beta}}^{\prime}\boldsymbol{x},j=1,2 $$

Where, *α*_*j*_ are separate intercept parameters, *j* is the level of an ordered category with 3 levels, ***β***^′^ different sets of regression parameters for each logit and ***x*** are a set of explanatory variables. The model thresholds and coefficients are estimated simultaneously using maximum likelihood procedure. Each cumulative logit has intercept, which increases with the categories of the outcome. The model assumes the same effects of ***β*** for each of the two dependent variables [33].

Crude and adjusted odds ratio with their 95% confidence intervals were calculated to determine the strength and presence of associations. We used “*svy*” set command in Stata to adjust for clustering effect due to the complex sampling design of the study at the village level. We test the proportionality of odds for the outcomes using likelihood ratio and Brant tests. All the analysis were done using Stata version 15 [34]. The STROBE guidelines for cross-sectional studies informed the design and reporting of this study [35].

## Results

### Socio-demographic characteristics of study participants

A total of 519 Kenyan and 409 Tanzanian women had a live birth(s) in the 2 years preceding the surveys. The overall median maternal age was 26 (IQR: 22–31) years with the same age distribution in the two countries. Seventy-nine per cent (*n* = 409/519) and 76.8% (*n* = 314/409) of the Kenyan and Tanzanian women delivered at a health facility respectively with a majority of the women attending at least four ANC visits during their last pregnancy (Table 1).

**Table 1:** Socio-demographic and obstetric history of women aged 15–49 years in Tanzania and Kenya

Variables	Kenya (***N*** = 519)	Tanzania (***N*** = 409)	Total (***N*** = 928)
**Maternal age, years**			
Median, IQR	26 (22–31)	27 (23–33)	27 (23–32)
15–19	51 (9.8)	34 (8.3)	85 (9.2)
20–29	288 (55.5)	214 (52.3)	502 (54.1)
30–29	148 (28.5)	134 (32.8)	282 (30.4)
40–49	32 (6.2)	27 (6.6)	59 (6.4)
**Marital status**			
Married	402 (77.5)	329 (80.4)	731 (78.77)
In-a-union	43 (8.3)	23 (5.6)	66 (7.1)
Not-in-union	74 (14.3)	57 (13.9)	131 (14.1)
**Level of education**			
No formal	93 (17.9)	56 (13.7)	149 (16.1)
Primary	293 (56.4)	273 (66.7)	566 (61.0)
Secondary+	133 (25.6)	80 (19.6)	213 (22.9)
**Place of delivery**			
Home/on the way	110 (21.2)	95 (23.2)	205 (22.1)
Health facility (Public/Private)	409 (78.8)	314 (76.8)	723 (77.9)
**Number of ANC visits**			
None /do not know	39 (7.5)	14 (3.4)	53 (5.7)
1–3 visits	181 (34.9)	161 (39.4)	342 (36.8)
4+ visits	299 (57.6)	234 (57.2)	533 (57.4)

*IQR* Interquartile range; *ANC* Antenatal care

### Birth preparedness and complication readiness

Overall, only 9.7% (*n* = 90) of the women were well-prepared while 35.7% (*n* = 331) were not prepared for their last birth with 11.4% (*n* = 59) Kenyan women being well-prepared compared to 7.6% (*n* = 31,) Tanzanian women. Setting aside funds for delivery services was the most common preparation made while identification of a blood donor before delivery was the least common preparation. Less than half of the women discussed the place of delivery (38.5%) or arranged for transport to a health facility (29.5%) and only a few (10.1%) discussed who will perform the delivery (Table 2).

**Table 2:** Level of BPCR for women aged 15–49 years in Kenya and Tanzania

BPCR components	Kenya	Tanzania	Total
Discussed place of delivery	129 (41.2)	101 (35.6)	230 (38.5)
Discussed who will perform delivery	40 (12.8)	20 (7.0)	60 (10.1)
Set aside funds for delivery	203 (64.9)	207 (72.9)	410 (68.7)
Arranged transport	116 (37.1)	60 (21.1)	176 (29.5)
Identified blood donor(s)	1 (0.3)	3 (1.1)	4 (0.7)
**Overall preparation**			
Not prepared (None mentioned)	206 (39.7)	125 (30.6)	331 (35.7)
Less prepared (< 3 out of 5)	254 (48.9)	253 (61.9)	507 (54.6)
Well-prepared (> 3 out of 5)	59 (11.4)	31 (7.6)	90 (9.7)

### Obstetric danger signs

Table 3 shows the level of knowledge on danger signs across the continuum of care among the respondent women. In both Kenya and Tanzania, few women had good knowledge about danger signs during pregnancy (21 and 17.8% respectively), labour and childbirth (13.9 and 15.2% respectively) and postpartum (13.7 and 10.0% respectively). A majority (60%) of women did not know danger signs in pregnancy, childbirth and postpartum.

**Table 3:** Knowledge of danger signs during pregnancy, labour and childbirths and post-partum among women 15–49 years in Tanzania and Kenya

Knowledge of danger signs^a^	Kenya (***N*** = 519)	Tanzania (***N*** = 409)	Total (***N*** = 928)
**During Pregnancy**			
No knowledge	286 (55.1)	195 (47.7)	481 (51.8)
Poor Knowledge	124 (23.9)	141 (34.5)	265 (28.6)
Good Knowledge	109 (21.0)	73 (17.8)	182 (19.6)
**During labour and childbirth**			
No knowledge	326 (62.8)	208 (50.9)	534 (57.5)
Poor Knowledge	121 (23.3)	139 (34.0)	260 (28.0)
Good Knowledge	72 (13.9)	62 (15.2)	134 (14.4)
**During postpartum**			
No knowledge	299 (57.6)	229 (56.0)	528 (56.9)
Poor Knowledge	149 (28.7)	139 (34.0)	288 (31.0)
Good Knowledge	71 (13.7)	41 (10.0)	112 (12.1)

^a^No knowledge (zero danger signs mentioned), poor knowledge (< 3 mentioned), good knowledge (> 3 mentioned)

The most commonly known danger sign during pregnancy, labour and childbirth and postpartum period was vaginal bleeding. Kenyan women were more knowledgeable about vaginal bleeding during pregnancy than Tanzanian women (62.7% versus 49.5%). This was also the case for heavy vaginal bleeding, however, the proportion of women mentioning severe vaginal bleeding (> 12 h) during labour and pregnancy as danger sign were similar for Kenya and Tanzania (Additional file 1).

### Factors associated with BPCR

Overall, education levels, knowledge of danger signs during postpartum and place of delivery were significantly associated with BPCR. Women with a secondary and higher level of education were better prepared for childbirth and its complications compared to women without formal education (aOR: 2.24; 95% CI: 1.39–3.59). Women who had delivered within a health facility had 1.63 times higher odds of preparation than those who had delivered out of health facility (aOR: 1.63; 95% CI: 1.15–2.29). Level of preparation increases with the level of knowledge of danger signs during the postpartum period; women with good knowledge of danger signs had 2.69 higher odds of preparation compared to those who were not aware of danger signs during postpartum (aOR: 2.69; 95% CI: 1.24–5.79). Women who attended four or more ANC visits had 42% higher odds of being well-prepared for birth and its complication (aOR: 1.42; 95% CI: 1.13–1.78) compared to those who attended less than four ANC visits. (Table 4).

**Table 4:** Proportional odds regression model on maternal socio-demographic and obstetric characteristics factors associated with the practices of birth preparedness and complication readiness in Kenya and Tanzania

Variables	Level of birth preparedness and complication readiness					
	Not prepared (***n*** = 331)	Less prepared (***n*** = 507)	Well prepared (***n*** = 90)	Crude Odds Ratio (95% CI)	Adjusted Odds Ratio (95% CI)	***p***-value
Maternal age	26 (23–32)†	27 (22–32)†	29 (23–34)†	1.02 (1.00–1.03)	1.01 (0.99–1.04)	0.157
**Marital status**						
Married	255 (77.0)	400 (78.9)	76 (84.4)	1	1	
In-a-union	20 (6.0)	39 (7.7)	7 (7.8)	1.18 (0.74–1.86)	1.18 (0.74–1.86)	0.477
Not-in-union	56 (17.0)	68 (13.4)	7 (7.8)	0.68 (0.49–0.94)*	0.75 (0.51–1.08)	0.118
**Level of education**						
No formal	73 (22.1)	68 (13.4)	8 (8.9)	1	1	
Primary	199 (60.1)	315 (62.1)	52 (57.8)	1.77 (1.24–2.52)*	1.59 (1.14–2.20)	0.007*
Secondary+	59 (17.8)	124 (24.5)	30 (33.3)	2.60 (1.68–4.03)*	2.24 (1.39–3.59)	0.002*
**Place of delivery**						
Home/on the way	93 (28.1)	104 (20.5)	8 (8.9)	1	1	
Health facility	238 (71.9)	403 (79.5)	82 (91.1)	1.82 (1.33–2.50)*	1.63 (1.15–2.29)	0.007*
**ANC attendance**						
None/1–3 visits	163 (49.3)	206 (40.6)	26 (28.9)	1	1	
4+ visits	168 (50.8)	301 (59.4)	64 (71.1)	1.60 (1.24–2.05)*	1.42 (1.13–1.78)	0.004*
**Danger signs during Pregnancy**						
No knowledge	223 (67.4)	218 (43.0)	40 (44.4)	1	1	
Poor knowledge	74 (22.4)	171 (33.7)	20 (22.2)	1.88 (1.42–2.48)*	1.20 (0.83–1.74)	0.309
Good knowledge	34 (10.3)	118 (23.3)	30 (33.4)	3.33 (2.54–4.37)*	1.43 (0.85–2.41)	0.174
**Danger signs during labour and childbirth**						
No knowledge	238 (71.9)	254 (50.1)	42 (46.7)	1	1	
Poor knowledge	68 (20.5)	171 (33.7)	21 (23.3)	1.91 (1.45–2.51)*	1.20 (0.87–1.65)	0.264
Good knowledge	25 (7.6)	82 (16.2)	27 (30.0)	3.55 (2.23–5.67)*	1.57 (0.93–2.67)	0.090
**Danger signs during postpartum**						
No knowledge	242 (73.1)	249 (49.1)	37 (41.1)	1	1	
Poor knowledge	71 (21.5)	188 (37.1)	29 (32.2)	2.28 (1.79–2.90)*	1.52 (1.06–2.18)	0.023*
Good knowledge	18 (5.4)	70 (13.8)	24 (26.7)	4.38 (2.51–7.63)*	2.69 (1.24–5.79)	0.014*

†Median (IQR); *Ref* Reference category; *significant *p*-value < 0.05; *IQR* Interquartile range; *ANC* Antenatal car

The country-specific analyses showed that BPCR was associated with a secondary and higher level of education, unmarried status, four or more ANC attendance, and poor knowledge of postpartum danger signs in Kenya (Additional file 2). In Tanzania, BPCR was associated with a primary and higher level of education, poor knowledge of pregnancy and labour/childbirth danger signs and good knowledge postpartum danger signs (Additional file 3).

## Discussion

We determined the magnitude and associated factors of BPCR among women of reproductive age who delivered within 2 years before the surveys in Kaloleni/Rabai and Bomachoge-Borabu sub-counties in Kenya and Mwanza region in Tanzania. Only a tenth of the women was well-prepared for birth in both countries with more Kenyan than Tanzanian women being well-prepared for birth (11.4% versus 7.6%). The overall and country-specific proportions of well preparedness were lower than facility-based studies in Rwanda (22.3%) [6], Uganda (28.3%) [8], West Pokot Kenya (28%) [7], community-based studies in Ethiopia (25.7–38.9%) [1, 9–12] and Chamwino district in Tanzania (58.4%) [13]. The low proportion of well-preparedness for birth could also be attributed to a low level of education and poor or inadequate counselling on BPCR during ANC attendance. More than half of the pregnant women who were unprepared or less prepared for birth attended at least four ANC sessions. Pregnant women are expected to be counselled on BPCR during ANC and are therefore more likely to be well-prepared for the birth and its complication. A third of well-prepared women had secondary and higher education levels compared to only 17.8% among those who were unprepared for birth. Kalisa and Malande found maternal education of secondary or higher levels was associated with being well-prepared [27]. To improve on BPCR, strategies such as male involvement [27, 36], community health workers [28, 37], pregnant mother conferences [11] and interactive mobile messaging alert system [38] could be adopted.

Similar to other BPCR studies, setting aside funds for delivery services was the most common preparation made by the women while identification of a blood donor before delivery was the least common preparation [2, 6, 12, 13, 39]. In our study, 65 and 73% of the Kenyan and Tanzanian women set aside funds respectively, which was lower than the 84.1% of women who set aside funds in central Tanzania [13], 87.5% in Rwanda [6] and 92.4% in Nigeria [40]. Central Tanzania hosts the county capital and is a high-income area, with better access to healthcare. The Kenyan rate was higher than the 12% in West Pokot [7]. West Pokot is a pastoralist community with poor access to health care and is one of the counties in Kenya with the highest maternal mortality.

Overall, the proportion of women who identified blood donors was lower than reported in other studies; 2.2% [7] to 25% [41], 8.2% in Ethiopia [1], 15% in Uganda [39], 17.5% in Tanzania [42] and 60.8% in South West Nigeria [40]. This could be explained by differences in the implementation of focused antenatal care and BPCR within a country and across countries [13].

Our study found that knowing any danger signs during the postpartum period was a significant factor of BPCR. Women with any knowledge of danger signs during postpartum had a higher likelihood to plan for the birth and its complication which is in line with the Ethiopian and Rwanda studies [2, 6]. Knowledge of any danger signs during pregnancy and childbirth were significantly associated with BPCR in Tanzania but not in Kenya. Women who have good knowledge of danger signs can seek treatment services in time without any delay in seeking maternal healthcare services [43]. Overall, knowledge of danger signs of pregnancy is a predictor of birth preparedness [44] and having some knowledge of the danger signs in pregnancy and childbirth is better than none. Knowledge of danger signs is important in the preparation of births and its complication and should be emphasized.

Delivering within a health facility was also associated with BPCR. More than three-quarters of the women delivered in health facilities but only less than half of them discussed the place of delivery or arranged transport to a health facility. Women who plan to deliver in a health facility are aware of the benefits of safe delivery and would know where to seek care. BPCR interventions contribute to an increase in the number of hospital deliveries [3, 13].

In this study, women with at least a primary education level were likely to know the importance of BPCR. Educated women tend to understand the benefits of early birth preparation, usually have autonomy in decision-making on their health and health of their child and can adhere to counselling sessions offered during the ANC visits. Similar findings were observed in studies conducted in Mpwapwa and Chamwimo districts of Tanzania [23, 45], Kenya [41] and Rwanda [27].

Women who attended four or more ANC visits had increased odds of being prepared for birth and its complication. This was in line with studies in Chamwimo district of Tanzania [45], Duguna Fango district of Ethiopia [20] and Makueni County, Kenya [41] that found attending ANC at least four times was significantly associated with the BPCR. In our study, though, most of the women who were both well-prepared and unprepared for birth attended at least four ANC sessions. During routine ANC, women are expected to be counselled on the danger signs during pregnancy, childbirth and labour and postnatal period and helped to plan their birth. In Kenya and Tanzania, health talks on BPCR are given to pregnant women during ANC visits in large groups before individualised ANC check-up. Therefore, only women in attendance at the time of the health talks benefit from a comprehensive discussion of components and importance of BPCR. Also, due to the large group counselling, some specific individual concerns may not be effectively addressed, and some information may be misinterpreted or missed by the mothers due to disruption or lack of concentration. This highlights the need to improve the quality of health education on BPCR to pregnant women and their families during ANC and through other forums such as chief’s forums (*barazas),* women interest groups [5] and community health workers [28].

In Kenya, not being in a union was associated with reduced odds of BPCR. Studies have shown that married women have higher odds of BPCR than unmarried women [46, 47]. For example, a hospital-based study in Kenya found married women to have 10 times higher odds of BPCR than unmarried women [47]. Birth preparedness among married women could be attributed to spousal and moral support before and during pregnancy and old age.

### Strengths and limitations

This study adds to the existing literature on preparation for BPCR and drivers on birth preparedness and level of knowledge of danger signs in East Africa. The study did not include other factors such as male involvement, household wealth, and access to and availability of health services that could have further explained BPCR. There could also be information bias as the questions were asked to all those who delivered in the last 2 years. Some might not have recalled the danger signs as taught during their ANC visits. Most of these questions had probes to help the respondents remember the appropriate responses. The data was collected among women of reproductive age in Kenya and Tanzania region, which limits the generalizability of our study findings to similar population groups in low resource settings.

## Conclusion

The level of well-prepared for BPCR and obstetric danger signs was low. Knowledge of danger signs during the postpartum period, delivery in health facility, antenatal care and at least primary education levels were associated with BPCR. Pregnant women should continuously be encouraged to attend ANC and deliver at a health facility. ANC sessions should be optimised to effectively provide knowledge on danger signs across the continuum of care and enhance BPCR. There is also a need for qualitative research among this population to explore further and verify some of these findings and explore interventions to enhance BPCR.

## Supplementary information

**Additional file 1.** Type of key obstetric danger signs spontaneously reported by Kenya and Tanzania respondents, respectively.

**Additional file 2 **Proportional odds regression model on maternal socio-demographic and obstetric characteristics factors associated with the practices of birth preparedness and complication readiness in **Kenya**, 2016.

**Additional file 3 **Proportional odds regression model on maternal socio-demographic and obstetric characteristics factors associated with the practices of birth preparedness and complication readiness in **Tanzania**, 2017.

## Data Availability

The datasets generated and analysed for this study are available on request and based on an official data transfer agreement at the Centre of Excellence in Women and Child Health East Africa at the Aga-Khan University.

## Abbreviations

ANC: Antenatal care
aOR: Adjusted odds ratio
AQCESS: Access to quality care through extending and strengthening health systems
BPCR: Birth preparedness and complication readiness
CI: Confidence interval
IMPACT: Improving access to reproductive, maternal and newborn health in Mwanza, Tanzania
IQR: Interquartile range
