# Supplementary material for: Birth preparedness and complication readiness among women of reproductive age in Kenya and Tanzania: a community-based cross-sectional survey
Source: BMC Pregnancy Childbirth. 2020 Oct 19;20:636. doi: 10.1186/s12884-020-03329-5 (PMC7574438; doi:10.1186/s12884-020-03329-5)
Supplement: Supplementary file 1 — Additional file 1. Type of key obstetric danger signs spontaneously reported by Kenya and Tanzania respondents, respectively. [file 12884_2020_3329_MOESM1_ESM.docx]

**Additional file 1**. Type of key obstetric danger signs spontaneously reported by Kenya and Tanzania respondents, respectively

| **Obstetric danger signs** | **Kenya** | **Tanzania** |
| --- | --- | --- |
|  | **n (%)** | **n (%)** |
| **Danger signs during pregnancy** | **233/519 (44.9)** | **214/409 (52.3)** |
| Difficulty breathing | 35 (15.0) | 17 (26.6) |
| Fatigue/tiredness | 56 (24.0) | 57 (26.6) |
| Vaginal bleeding | 146 (62.7) | 106 (49.5) |
| Baby not moving | 65 (27.9) | 33 (15.4) |
| Loss of consciousness | 36 (15.5) | 27 (12.6) |
| Convulsions | 43 (18.5) | 21 (9.8) |
| Headache and blurred vision | 57 (24.5) | 55 (25.7) |
| Early labour pain before term | 57 (15.9) | 29 (13.6) |
| Vaginal discharge | 40 (17.2) | 25 (11.7) |
| Signs of malaria (feeling cold, fever, vomiting) | 36 (15.5) | 31 (14.5) |
| High blood pressure | 26 (11.2) | 29 (23.6) |
| Swelling of face and arms | 24 (10.3) | 44 (20.6) |
| **Danger signs during labour and childbirth** | **193/519 (37.2)** | **201/409 (49.1)** |
| Severe vaginal bleeding (>12 hours) | 88 (45.6) | 90 (44.8) |
| Prolonged labour | 79 (40.9) | 69 (34.3) |
| Convulsions | 46 (23.8) | 17 (8.5) |
| Retained placenta | 57 (29.5) | 19 (9.5) |
| Baby lying sideways or upside down | 57 (29.5) | 38 (18.9) |
| Fever | 32 (16.6) | 38 (19.4) |
| Green or brown waters | 17 (8.8) | 27 (13.4) |
| Strong headache | 35 (18.2) | 30 (14.9) |
| Blurred or double vision | 15 (7.8) | 10 (5.0) |
| Sudden, steady severe pain at the top of the belly | 8 (4.2) | 37 (18.4) |
| Overactive reflexes | 6 (3.1) | 5 (2.5) |
| High blood pressure | 30 (15.5) | 27 (13.4) |
| Protein in urine | 2 (1.0) | 7 (3.5) |
| Does not know any of the above | 12 (6.2) | 5 (2.5) |
| **Danger signs during postpartum period** | **220/519 (42.4)** | **180/409 (44.0)** |
| High fever, lower abdominal pain, or foul-smelling discharge (infection) | 82 (37.3) | 75 (41.7) |
| Severe headache, blurred vision, high blood pressure | 75 (34.1) | 44 (24.4) |
| Convulsions or fits (eclampsia) | 65 (29.6) | 26 (14.4) |
| Heavy vaginal bleeding | 137 (62.3) | 85 (47.2) |
| Urinary or faecal incontinence (obstetric fistula) | 17 (7.7) | 19 (10.6) |
| Extreme tiredness, anaemia | 49 (22.3) | 33 (18.3) |
| Anxiety or depression (puerperal psychosis) | 6 (2.7) | 9 (5.0) |
| Breast problems (engorgement, sore, cracked bleeding, or inverted nipples) | 16 (7.3) | 16 (8.9) |
