# Supplementary material for: Birth preparedness and complication readiness among women of reproductive age in Kenya and Tanzania: a community-based cross-sectional survey
Source: BMC Pregnancy Childbirth. 2020 Oct 19;20:636. doi: 10.1186/s12884-020-03329-5 (PMC7574438; doi:10.1186/s12884-020-03329-5)
Supplement: Supplementary file 2 — Additional file 2 Proportional odds regression model on maternal socio-demographic and obstetric characteristics factors associated with the practices of birth preparedness and complication readiness in Kenya, 2016. [file 12884_2020_3329_MOESM2_ESM.docx]

**Additional file 2**. Proportional odds regression model on maternal socio-demographic and obstetric characteristics factors associated with the practices of birth preparedness and complication readiness in **Kenya**, 2016

| **Variables** | **Not prepared (n=125)** | **Less prepared (n=253)** | **Well-prepared (n=31)** | **Crude odd ratio (95% CI)** | **p-value** | **Adjusted odd ratio (95% CI)** | **p-value** |
| --- | --- | --- | --- | --- | --- | --- | --- |
| Maternal age | 26 (23–31) | 26 (22–30) | 29 (22–34) | 1.01 (0.98–1.03) | 0.658 | 1.01 (0.98–1.04) | 0.572 |
| **Marital status** |  |  |  |  |  |  |  |
| Married | 157 (76.2) | 193 (76.0) | 52 (88.1) | 1 |  | 1 |  |
| In-a-union | 11 (5.3) | 28 (11.0) | 4 (6.8) | 1.39 (0.86–2.25) | 0.171 | 1.32 (0.76–2.28) | 0.310 |
| Not in union | 38 (18.5) | 33 (13.0) | 3 (5.1) | 0.55 (0.38–0.80) | 0.003* | 0.60 (0.39–0.93) | 0.024* |
| **Level of education** |  |  |  |  |  |  |  |
| No formal | 48 (23.3) | 39 (13.4) | 6 (10.2) | 1 |  | 1 |  |
| Primary | 117 (56.8) | 141 (55.5) | 35 (59.3) | 1.66 (1.05–2.63) | 0.032* | 1.48 (0.91–2.42) | 0.114 |
| Secondary+ | 41 (19.9) | 74 (29.1) | 18 (30.5) | 2.31 (1.34–3.96) | 0.004* | 1.99 (1.06–3.74) | 0.034* |
| **Place of delivery** |  |  |  |  |  |  |  |
| Home/on the way | 52 (25.2) | 54 (21.3) | 4 (6.0) | 1 |  | 1 |  |
| Health facility | 154 (74.8) | 200 (78.7) | 55 (93.2) | 1.68(1.15-2.44) | 0.008* | 1.50 (0.92–2.43) | 0.098 |
| **ANC attendance** |  |  |  |  |  |  |  |
| None/1–3 visits | 104 (50.5) | 99 (39.0) | 17 (28.8) | 1 |  | 1 |  |
| 4+ visits | 102 (49.5) | 155 (61.0) | 42 (71.2) | 1.77 (1.35–2.33) | <0.001** | 1.63 (1.24–2.14) | 0.001* |
| **Pregnancy danger signs** |  |  |  |  |  |  |  |
| Not aware | 137 (66.5) | 117 (46.1) | 32 (54.2) | 1 |  | 1 |  |
| Poor knowledge | 47 (22.8) | 71 (27.9) | 6 (10.2) | 1.22 (0.82–1.82) | 0.305 | 0.88 (0.51–1.50) | 0.621 |
| Good knowledge | 22 (10.7) | 66 (26.0) | 21 (35.6) | 3.02 (2.06–4.43) | <0.0001** | 1.34 (0.62–2.92) | 0.445 |
| **Labour and childbirth danger signs** | |  |  |  |  |  |  |
| Not aware | 149 (72.3) | 145 (57.1) | 32 (54.2) | 1 |  | 1 |  |
| Poor knowledge | 45 (21.8) | 66 (26.0) | 10 (17.0) | 1.28 (0.77–2.14) | 0.326 | 0.88 (0.47–1.63) | 0.665 |
| Good knowledge | 12 (5.8) | 43 (16.9) | 17 (28.8) | 3.62 (2.11–6.23) | <0.0001** | 1.42 (0.66–3.04) | 0.358 |
| **Postpartum danger signs** |  |  |  |  |  |  |  |
| Not aware | 145 (70.4) | 128 (50.4) | 26 (44.1) | 1 |  | 1 |  |
| Poor knowledge | 46 (22.3) | 88 (34.6) | 15 (25.4) | 1.85 (1.33–2.56) | 0.001* | 1.49 (1.02–2.19) | 0.041* |
| Good knowledge | 15 (7.3) | 38 (15.0) | 18 (30.5) | 3.81 (1.96–7.41) | <0.0001** | 2.36 (0.97–5.70) | 0.058 |

COR: Crude Odds Ratio; AOR: Adjusted Odds Ratio; ANC: Antenatal care; *p <0.05; **p < 0.001
