# Supplementary material for: Birth preparedness and complication readiness among women of reproductive age in Kenya and Tanzania: a community-based cross-sectional survey
Source: BMC Pregnancy Childbirth. 2020 Oct 19;20:636. doi: 10.1186/s12884-020-03329-5 (PMC7574438; doi:10.1186/s12884-020-03329-5)
Supplement: Supplementary file 3 — Additional file 3 Proportional odds regression model on maternal socio-demographic and obstetric characteristics factors associated with the practices of birth preparedness and complication readiness in Tanzania, 2017. [file 12884_2020_3329_MOESM3_ESM.docx]

**Additional file 3**. Proportional odds regression model on maternal socio-demographic and obstetric characteristics factors associated with the practices of birth preparedness and complication readiness in **Tanzania**, 2017

| **Variables** | **Not prepared (n=125)** | **Less prepared (n=253)** | **Well-prepared (n=31)** | **Crude odd ratio (95% CI)** | **p-value** | **Adjusted odd ratio (95% CI)** | **p-value** |
| --- | --- | --- | --- | --- | --- | --- | --- |
| **Maternal age** | 27 (22–33) | 28 (23–33) | 30 (23–37) | 1.03 (1.00–1.06) | 0.041 | 1.03 (0.99–1.06) | 0.055 |
| **Marital status** |  |  |  |  |  |  |  |
| Married | 98 (78.4) | 207 (81.8) | 24 (77.4) | 1 |  | 1 |  |
| In-a-union | 9 (7.2) | 11 (4.4) | 3 (9.7) | 0.82 (0.28–2.41) | 0.706 | 0.99 (0.36–2.79) | 0.999 |
| Not-in-union | 28 (14.4) | 35 (13.8) | 4 (12.9) | 0.93 (0.57–1.50) | 0.750 | 1.05 (0.58–1.89) | 0.870 |
| **Level of education** |  |  |  |  |  |  |  |
| No formal | 25 (20.0) | 29 (11.4) | 2 (6.5) | 1 |  | 1 |  |
| Primary | 82 (65.6) | 174 (68.8) | 17 (54.8) | 1.84 (1.25–2.72) | 0.003* | 2.00 (1.41–2.85) | <0.0001** |
| Secondary+ | 18 (14.4) | 50 (19.8) | 12 (38.7) | 3.30 (1.60–6.83) | 0.002* | 3.55 (1.98–6.27) | <0.0001** |
| **Place of delivery** |  |  |  |  |  |  |  |
| Home/on the way | 41 (32.8) | 50 (19.8) | 4 (12.9) | 1 |  | 1 |  |
| Health facility | 84 (67.2) | 203 (80.2) | 27 (87.1) | 2.09 (1.25–3.49) | 0.007* | 1.73 (1.06–2.84) | 0.031 |
| **ANC attendance** |  |  |  |  |  |  |  |
| None/1–3 visits | 59 (47.2) | 107 (42.3) | 9 (29.0) | 1 |  | 1 |  |
| 4+ visits | 66 (52.8) | 146 (57.7) | 22 (71.0) | 1.39 (0.90–2.15) | 0.135 | 1.24 (0.82–1.86) | 0.293 |
| **Pregnancy danger signs** |  |  |  |  |  |  |  |
| Not aware | 86 (68.8) | 101 (39.9) | 8 (25.8) | 1 |  | 1 |  |
| Poor knowledge | 27 (21.6) | 100 (39.5) | 14 (45.2) | 3.18 (2.02–5.02) | <0.0001** | 1.84 (1.16–2.90) | 0.011* |
| Good knowledge | 12 (9.6) | 52 (20.6) | 9 (29.0) | 3.90 (2.36–6.42) | <0.0001** | 1.57 (0.93–2.65) | 0.086 |
| **Labour and childbirth danger signs** |  |  |  |  |  |  |  |
| Not aware | 89 (71.2) | 109 (43.1) | 10 (32.3) | 1 |  | 1 |  |
| Poor knowledge | 23 (18.4) | 105 (41.5) | 11 (35.5) | 3.06 (1.82–5.12) | <0.0001** | 1.78 (1.05–3.01) | 0.034 |
| Good knowledge | 13 (10.4) | 39 (15.4) | 10 (32.3) | 3.48 (2.02–6.00) | <0.0001** | 1.63 (0.92–2.89) | 0.092 |
| **Postpartum danger signs** |  |  |  |  |  |  |  |
| Not aware | 97 (77.6) | 121 (47.8) | 11 (35.5) | 1 |  | 1 |  |
| Poor knowledge | 25 (20.0) | 100 (39.5) | 14 (45.2) | 3.14 (1.90–5.19) | <0.0001** | 1.56 (0.89–2.75) | 0.114 |
| Good knowledge | 3 (2.4) | 32 (12.7) | 6 (19.4) | 5.77 (2.83–11.8) | <0.0001** | 3.48 (1.48–8.18) | 0.006* |

COR: Crude Odds Ratio; AOR: Adjusted Odds Ratio; ANC: Antenatal care; *p <0.05; **p < 0.001
